# Supporting a Different Type of Death: Experiences of Family Members of Recipients of Medical Assistance in Dying

**DOI:** 10.1002/nop2.70843

**Published:** 2026-09-29

**Authors:** Tracy L. Powell, Kelli Stajduhar, Michael Prince, Sally Thorne

**Affiliations:** ^1^ School of Nursing and Midwifery Mount Royal University Calgary Alberta Canada; ^2^ School of Nursing The University of Victoria Victoria British Columbia Canada; ^3^ Professor Emeritus The University of Victoria Victoria British Columbia Canada; ^4^ School of Nursing The University of British Columbia Vancouver British Columbia Canada

## Abstract

**Aim:**

To explore the experiences of family members when a relative or close person receives medical assistance in dying.

**Design:**

Guided by Interpretive Description methodology, this qualitative study was conducted across two settings. Participants included bereaved family members of individuals who received clinician‐assisted medical assistance in dying when natural death was reasonably foreseeable and key informants connected to medical assistance in dying, palliative care and end‐of‐life organizations.

**Methods:**

Data were collected between June 2020 and February 2021 through demographic surveys, in‐depth interviews, documents and artifacts, field notes and analytic memos. Data collection and analysis occurred concurrently and iteratively, supporting the development of practice‐relevant interpretations of family members' experiences.

**Results:**

Forty‐six participants, including 31 bereaved family members and 15 key informants, participated. Three interpretive themes characterized family members' experiences: (a) ‘They want medical assistance in dying, now what?’, (b) ‘Prepared but maybe not ready’ and (c) ‘An evolving understanding about this type of death’. Family members described navigating healthcare processes, assuming practical and emotional responsibilities, and negotiating their own beliefs while supporting the individual's decision. They also described meaningful opportunities for connection and farewell alongside uncertainty, moral strain and emotional upheaval. Supporting a relative's decision to pursue medical assistance in dying did not necessarily correspond to emotional preparedness for the death or subsequent bereavement.

**Conclusion:**

Medical assistance in dying is experienced not only as an individual decision but also as a relational process with emotional, practical and moral implications for family members. A family‐in‐focus approach may complement person‐centred care by increasing the visibility of family members throughout the MAiD trajectory. Proactive communication, recognition of emotional complexity, and access to psychosocial and bereavement support may better address the needs of family members while maintaining the autonomy of the individual accessing an assisted death.

**Patient or Public Contribution:**

No Patient or Public Contribution.

## 1 Introduction

The death of a relative or other close individual can profoundly affect families, who must navigate both emotional upheaval and the practical demands of healthcare and end‐of‐life care. Although research has addressed caregiving, caregiver burden and bereavement (Handique et al. 2025), these findings may not fully apply to medical assistance in dying (MAiD), given its distinct ethical, legislative and clinical considerations. Although research on family members' perspectives on MAiD is expanding, less is known about how healthcare systems, jurisdictional implementation, and care processes influence their experiences before, during and after MAiD (Beuthin et al. 2021; Brown et al. 2020; Close et al. 2023). Such knowledge is critical for guiding nursing care for individuals accessing MAiD and their accompanying family members.

This paper presents findings from a qualitative Interpretive Description study exploring bereaved family members' perspectives following the MAiD‐related death of a relative or other close individual during the first 5 years of legalization in Canada (2016–2020). The study offers practice‐relevant insights into how contextual factors influence family members' experiences throughout the MAiD process. The analysis positions family members as actively engaged in preparing for, responding to, and reflecting on MAiD within relational, ethical and healthcare contexts. These findings inform a family‐in‐focus approach to nursing care and identify opportunities to enhance communication, continuity of care and family support through integrated MAiD care and supportive policy.

## 2 Background

The emotional, psychological and logistical burdens experienced by family caregivers of individuals with life‐limiting illness are well documented in palliative and end‐of‐life literature (Arnup 2018; Statistics Canada 2018). Family members often assume substantial practical and caregiving responsibilities with limited formal support, contributing to stress and unmet needs (Arnup 2018; National Alliance for Caregiving 2009). Following a death, they may continue to experience challenges related to role adjustment and physical and mental health (Statistics Canada 2018).

Emerging research suggests that family members' experiences in the context of assisted dying can involve moral tension, isolation and secrecy (Rumsey et al. 2026; Serota et al. 2024). Research from Switzerland, Belgium, the Netherlands and Oregon also identifies moral dilemmas and diverse bereavement experiences (Gamondi et al. 2018). However, these findings arise within legal and sociocultural contexts that differ from Canada.

Since Canada legalized MAiD in 2016 for individuals with medical conditions leading to a foreseeable natural death, research on family members' experiences has increased but remains limited. Canadian studies have documented the emotional complexity of supporting a relative through MAiD, as well as concerns related to privacy, legislative requirements, and variations in care and support across regions, organizations and settings (Crumley et al. 2023; Hales et al. 2019; Hashemi et al. 2021; Holmes et al. 2018; Oczkowski et al. 2021). Less is known about how MAiD implementation, healthcare processes, and models of care shape family members' experiences across the assisted dying trajectory. This gap may limit nurses' ability to anticipate and respond to family members' informational, emotional and relational needs.

Recognizing the central role of family members in end‐of‐life care and the evolving MAiD landscape in Canada, this study explored bereaved family members' experiences across two provincial settings. The study addressed a gap in understanding how jurisdictional policies, MAiD implementation, and healthcare processes influence these experiences, generating evidence to inform family‐in‐focus nursing care and healthcare policy.

## 3 The Study

### 3.1 Study Aims

Our study explored the research question: What are the experiences and perspectives of bereaved family members whose relative or other close person received MAiD? We aimed to (1) describe family members' experiences, including moral and ethical tensions; (2) understand how MAiD legislation and its implementation influenced these experiences; and (3) generate clinically meaningful, practice‐and policy‐relevant insights to inform support for family members.

## 4 Methodology

### 4.1 Design

We employed an Interpretive Description approach (Thorne 2016) to examine the experiences of bereaved family members of MAiD. This methodology is well suited to applied health research because it supports exploration of complex clinical and relational phenomena while developing practice‐relevant knowledge. Consistent with Interpretive Description, data collection and analysis occurred concurrently and iteratively, supporting the development and refinement of interpretations across individual, organizational and health system contexts. This approach enabled us to interpret bereaved family members' experiences within broader social, ethical, clinical and structural contexts. Key informants comprised the secondary participant group whose clinical, organizational, and system‐level perspectives provided contextual insight for interpreting family members' accounts. This approach moved beyond description to develop clinically meaningful insights relevant to MAiD teams, palliative care providers, nurses and other healthcare professionals, administrators and policymakers.

### 4.2 Theoretical Framework

Relational ethics guided our exploration of bereaved family members' experiences. Bergum and Dossetor (2005) describe relational ethics as emphasizing interconnectedness and framing experience as relational, situated and ethically complex. This focus on relationships and context supported our examination of experiences within the complex healthcare, legal and familial dimensions of MAiD. Relational ethics also challenges the view of autonomy by recognizing autonomy as socially embedded (Austin et al. 2003). In this study, relational ethics served as a sensitizing lens rather than a framework that predetermined analytic categories or interpretations. Interpretive Description guided the development of practice‐oriented understandings grounded in participants' accounts, while relational ethics directed attention to the relational and contextual aspects of their experiences.

### 4.3 Participants and Recruitment

Participant recruitment occurred between June 2020 and February 2021 and included two groups: (1) bereaved family members and (2) key informants. We recruited bereaved family members through social media (e.g., Facebook, X/Twitter) and direct outreach to organizations likely to have contact with this population, such as local Dying with Dignity Chapters, Wellspring Alberta, and the Institute on Aging and Lifelong Health at the University of Victoria. As recruitment progressed, the team also used nominated sampling. Thirty‐four individuals expressed interest in participating by contacting the research team; 32 met the eligibility criteria and provided informed consent. One individual subsequently withdrew before data collection without providing a reason. Given the lack of consensus on an appropriate interval for engaging bereaved individuals in research (Bentley and O'Connor 2015), we did not impose a minimum bereavement interval. Participation was determined by self‐assessed readiness.

We recruited key informants through purposive sampling to capture diverse professional perspectives about MAiD, palliative care and end‐of‐life care. As the study progressed, we used nominated and theoretical sampling to recruit individuals who could contribute additional clinical, organizational and system‐level perspectives. Potential participants received a recruitment letter describing the study and inviting them to contact the research team. Sixteen key informants consented to participate; one withdrew consent before data collection, citing workload demands.

### 4.4 Participants and Sampling

Interpretive Description acknowledges the complexity and diversity of human experience and seeks to generate clinically meaningful, practice‐oriented knowledge (Thorne 2016). Consistent with this methodology, we did not use theoretical or data saturation as a criterion for determining sample size. Our initial recruitment aim was 15–20 bereaved family members and 10–15 key informants per setting (Sandelowski 1995).

Data collection and analysis occurred concurrently and iteratively, enabling the research team to assess the richness and variation of participants' accounts and inform ongoing recruitment and analytic decisions. Recruitment concluded when the research team determined that the data demonstrated sufficient depth and variation to support interpretation and address the study aims (Thorne 2016). The objective was to understand factors influencing bereaved family members' experiences across contexts, rather than to compare the two settings.

### 4.5 Study Participants

The final sample comprised 46 participants: 31 bereaved family members as the primary participant group and 15 key informants as the secondary participant group.

### 4.6 Inclusion and Exclusion Criteria

#### 4.6.1 Bereaved Family Members

Eligible participants had to be at least 19 years old, able to speak and read English and able to provide informed consent. Residency criteria included living on Vancouver Island, elsewhere in British Columbia, or in the Calgary, South, and Central zones of Alberta, or elsewhere in Alberta. Participants needed to be family members of individuals who received clinician‐administered MAiD due to a reasonably foreseeable natural death, in either British Columbia or Alberta. Individuals whose relative or other close person received self‐administered MAiD were not eligible. The study focused on clinician‐administered MAiD because self‐administered MAiD is uncommon and involves distinct relational and experiential circumstances (Health Canada 2024).

#### 4.6.2 Key Informants

Key informants had to be at least 19 years old, able to speak and read English and capable of providing informed consent. We purposively selected individuals with professional experience in MAiD policy, practice, education, implementation, palliative care, or end‐of‐life services in the specified regions (Vancouver Island, British Columbia and the province of Alberta). Their perspectives offered contextual insight into the clinical, organizational, system‐level and relational factors influencing family members' experiences of MAiD.

### 4.7 Data Collection

The primary interviewer was a female Registered Nurse (RN), PhD candidate and Associate Professor in a School of Nursing at a Canadian university during the study period. She had clinical experience in oncology, palliative care and end‐of‐life care, as well as expertise in conducting qualitative, in‐depth interviews about sensitive topics. She had no prior clinical or personal relationship with participants. Participants were informed of her professional and academic role and the research purpose. The interviewer acknowledged that her clinical and academic background, along with her interest in family members' experiences of MAiD, could influence the research process and interpretation. To address this, she maintained reflexive field notes and analytic memos throughout data collection and analysis to critically examine her assumptions, positioning and potential influence.

Before data collection, all participants provided informed consent after receiving detailed information about the study purpose, procedures, potential risks and benefits, voluntary participation, and their right to withdraw without consequence. We used multiple data sources and strategies (a–d) to develop an understanding of bereaved family members' experiences. (a) We distributed group‐specific demographic questionnaires and collected responses by email. These questionnaires gathered demographic and participant‐specific information. The bereaved family member questionnaires also collected information about the MAiD event and the individual who received MAiD, including underlying medical condition, location of death and involvement of palliative care. Two family members reported two separate MAiD events, resulting in 33 demographic surveys.

(b) We conducted one in‐depth, open‐ended interview with each participant; we did not conduct repeat interviews. The research team developed separate interview guides for each participant group, and participants received the relevant guide before their interview (Tables 1 and 2). Although the guides were not formally pilot‐tested, we reviewed and refined them before data collection to align with the study aims and Interpretive Description methodology. Participants selected either Zoom or telephone for their interviews (*n* = 46), and only the participant and the interviewer were present. All interviews were audio‐recorded and transcribed verbatim by a professional transcriptionist.

**TABLE 1:** Interview guide (bereaved family member participants).

Can you tell me a little bit about yourself and how MAiD came to be part of your experience?
2 Can you describe a bit more about your specific experience as your person went through the MAiD process? (Prompt: focus on their level of involvement and responsibility in the process; if there were any tensions among others in their circle when MAiD was chosen; the most challenging part of their experience; the most beneficial part; discuss any support they received)
3 Can you tell me about your perspectives on your person's choice for MAiD? (Prompt: any moral and ethical issues; ask about their perspective on death because of the choice for MAiD)
4 Can you tell me about the MAiD process and how it impacted your experience? (Prompt: impression of the MAiD process as they experienced it; the location of the death and if they felt this impacted their experience; experience with health care professionals and the MAiD team; to what extent were palliative care services involved)
5 Can you tell me about your experience since your person's death? (Prompt: life since going through MAiD with the person who received MAiD; how was the death disclosed to family, friends and members of the community; what would they have liked or changed related to supports and services that they received)

**TABLE 2:** Interview guide (key informant participants).

To begin, please tell me a little bit about yourself and your involvement with MAiD
2 What has been your experience with family members in relation to MAiD?
3 What are the kinds of things that you have identified as impacting family members in relation to MAiD? What could enhance or ease their experience?
4 Do your colleagues ever discuss issues related to family members and MAiD? If so, how have they come about? Could you comment generally on the discussions?
5 From your perspective, are MAiD processes in your area being enacted as outlined in legislation and policy? What do you think could be improved?
6 Are there any documents you know of that pertain to what you have discussed and would be willing to share with me?

(c) We included 36 documents, including MAiD legislation, implementation guidelines, policies and processes, to provide context for participants' experiences. We reviewed these documents alongside participant accounts throughout analysis to situate interpretations within broader clinical, organizational and policy contexts. We also included 34 artifacts, such as journal entries and emails, that bereaved family members as meaningful during their relative's journey toward MAiD (Figure 1). This provided additional contextual and temporal insights.

**FIGURE 1:**
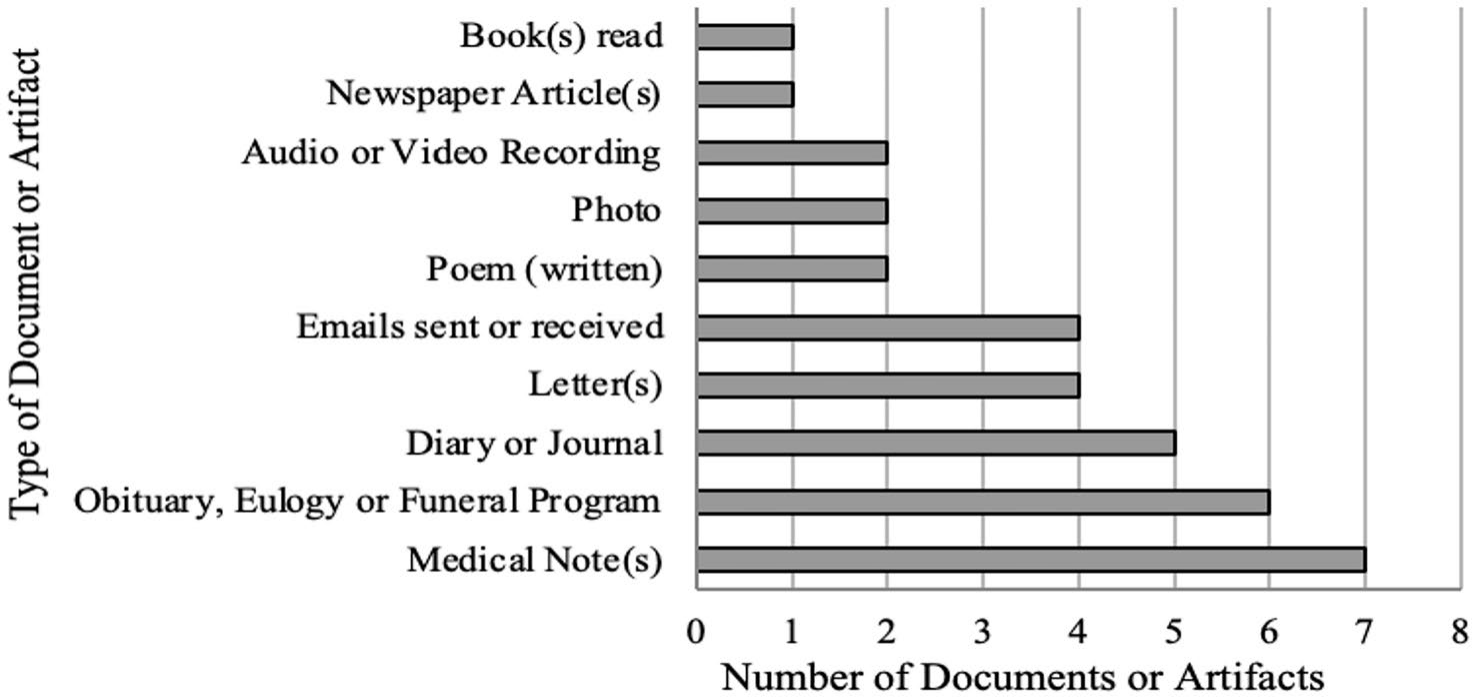
Type and number of personal documents and artifacts submitted by bereaved family member participants.

Interviews lasted between 45 min and 3 h. (d) The interviewer documented field notes and analytic memos for each interview (*n* = 46), recording methodological decisions, developing interpretations and reflexive observations. Rather than using data saturation as a predetermined endpoint, we assessed analytic adequacy iteratively throughout data collection. We considered the depth, breadth and variation of participants' accounts and whether additional interviews contributed to, challenged, or refined developing interpretations. The study adhered to ethical guidelines to protect participant privacy and confidentiality. We used Microsoft Excel and NVivo to organize and manage study data.

### 4.8 Data Analysis

The data analysis followed the principles of Interpretive Description, which prioritizes clinically meaningful, practice‐oriented knowledge. Data collection and analysis were conducted concurrently and iteratively, allowing for the refinement of interview questions and ongoing methodological reflection (Thorne 2016). NVivo supported qualitative data analysis, whereas Excel was used to analyze demographic survey data.

Morse's (1994) framework of comprehending, synthesizing, theorizing and reconceptualizing informed the analytic approach. Analysis began with the first interview and continued throughout data collection. The initial phase involved comprehending and synthesizing interview transcripts, analytic memos, field notes, documents and artifacts. Transcripts were reviewed for accuracy before analysis. The primary analytic focus was on bereaved family members' accounts, which informed both coding and interpretation. Key informant data were analyzed concurrently to provide clinical, organizational and system‐level context for interpreting family members' experiences. The team systematically considered convergence and divergence across participant accounts and data sources. We revisited documents and artifacts alongside interview data to situate our interpretations within broader social, ethical, clinical and policy contexts.

We organized data into broad, provisional categories and developed codes inductively rather than using predetermined codes or a coding tree (Thorne 2016). The primary researcher conducted and led the interpretive analysis. In total, five researchers were involved in data analysis, including the primary researcher. The team coded iteratively, continually reviewing, regrouping, refining and reconceptualizing codes and categories. The team examined relationships across participant perspectives, data sources and contexts through repeated cycles of coding, un‐coding, ungrouping and refinement (Patton 2015). The research team met to critically reflect on analytic decisions, while reflexive journaling and analytic memos document assumptions, positionality and interpretive shifts (Thorne 2016). These processes established an audit trail and enhanced analytic credibility. We remained attentive to contradictory and unexpected accounts throughout the interpretive process. Participants were not involved in reviewing or providing feedback on the findings, and we did not return interview transcripts to participants for comment or verification.

As analysis progressed toward reconceptualization, the research team examined how interpretations extended or challenged existing understandings of family members' experiences with MAiD (Thorne 2016). Rather than stopping at thematic description, the team considered relationships and meanings across individual, organizational and system contexts. This included the influence of social and structural forces such as power relations, policies, practices and relationships (Krawczyk et al. 2019; Wright et al. 2018). Guided by relational ethics, this multilevel interpretation informed clinically relevant, practice‐oriented insights into bereaved family members' experiences with MAiD.

### 4.9 Ethical Considerations

Our research received ethical approval from the Harmonized University of Victoria Health Research Ethics Board (REB), the Health Research Ethics Board of Alberta‐Community Health Committee (HREBA‐CHC) and the Human Research Ethics Board at Mount Royal University. We obtained informed consent from all participants and reminded them that they could withdraw or skip questions at any time. Participants received no financial compensation or other incentives. After each interview, participants received a resource document and we contacted them within 5 days to check in. We maintained confidentiality using pseudonyms, password‐protected files and secure storage. For Zoom interviews, we followed privacy protocols including turning off certain features (the ‘join before host’ function) and offering anonymity options. We securely transferred audio files to a professional transcriptionist, who removed identifying information. We destroyed recorded data after transcript verification; hard copies will be securely stored at Mount Royal University for 5 years before destruction.

### 4.10 Rigour

To support rigour, we drew on Thorne's (2016) four criteria for Interpretive Description: epistemological integrity, representative credibility, analytic logic and interpretive authority. Epistemological integrity was supported by alignment among the research question, methodology, sampling, data collection and interpretation. Using multiple data sources and incorporating diverse participant perspectives provided contextual depth and variation. Representative credibility was supported by linking interpretations to participants' accounts, revisiting transcripts throughout analysis, and attending to both convergent and divergent accounts.

Analytic logic was supported through an iterative audit trail documenting methodological and analytic decisions. Reflexive journaling and team discussions enhanced interpretive authority by critically examining assumptions, alternative interpretations and analytic choices. Detailed interview excerpts enabled readers to trace interpretations to participants' accounts and supported the credibility of the findings.

## 5 Findings

### 5.1 Description of Participants

#### 5.1.1 Bereaved Family Members

Thirty‐one bereaved family members participated, with 19 from Alberta and 12 from British Columbia. Ages ranged from 28 to 81 years (*M* = 63.06, SD = 13.99), and most were female (*n* = 26). Participants represented varied educational, cultural and relational backgrounds; daughters (*n* = 12) and wives (*n* = 8) were the most common relationships to the person who received MAiD. Most participants were retired at the time of the MAiD event (*n* = 18). Table 3 presents characteristics of bereaved family member participants.

**TABLE 3:** Characteristics of bereaved family member participants.

	Frequency
**Residency of the participant (*n =* 31)**	
Alberta	19
British Columbia	12
**Gender (*n =* 31)**	
Female	26
Male	5
**Ethnic or cultural background (*n =* 31)**	
Canadian/Caucasian/white	20
European	3
European Canadian	3
Chinese	1
Croatian	1
Jewish	1
German Canadian	1
Métis	1
**Highest degree of schooling (*n =* 31)**	
Baccalaureate degree	12
Post‐graduate degree	8
College diploma	5
High school diploma	4
Below high school	2
**Marital status (*n =* 31)**	
Widowed	14
Married	11
Common‐law	3
Single	1
Separated	1
Engaged	1
**Relationship to the deceased (*n =* 33)** ^a^	
Daughter	12
Wife	8
Sister	5
Son	4
Mother	1
Husband	1
Sister‐in‐law	1
Best friend	1
**Employment status when loved one had MAID event (*n =* 33)** ^a^	
Retired	18
Full‐time employment	8
Part‐time employment	3
Self‐employed	3
Casual/contract	1
Unemployed	1
Semi‐retired	1
**Current employment status (*n =* 31)**	
Retired	18
Full‐time employment	6
Part‐time employment	3
Self‐employed	1
Casual/contract	1
Unemployed	1
Semi‐retired	1

*Note:* This table outlines the characteristics of the sample of bereaved family members.

^a^ Two participants had two relatives who received MAiD.

We also collected information on the MAiD recipients and circumstances surrounding their deaths. Of the 33 MAiD deaths, 20 occurred in Alberta and 13 in British Columbia. Most recipients lived in the same city as the family member participant, and 10 shared the same home. Palliative care was involved in 23 cases. Cancer (*n* = 18) and neurological conditions (*n* = 10) were the most common diagnoses. Most MAiD events occurred at home (*n* = 20), and the recipient chose the location in 27 cases. In six cases, the recipient did not choose the location; and one was related to care needs. Physicians and nurses were the healthcare providers most commonly present at the provision.

#### 5.1.2 Key Informants

Fifteen key informants (13 female, 2 male) participated, ranging in age from 33 to 75 years (*M* = 51.2, SD = 11.5). Participants included registered nurses (*n* = 5), physicians (*n* = 4), nurse practitioners (*n* = 2), social workers (*n* = 2), a spiritual health practitioner and a licensed practical nurse. Eight worked exclusively on MAiD teams, four worked in both MAiD and palliative care, two worked in psychosocial support, and one worked in hospice care. Most held postgraduate (*n* = 7) or baccalaureate degrees (*n* = 4). Key informants were based in Alberta (*n* = 9) and British Columbia (*n* = 6), and most had been involved with MAiD since, or before, its implementation in June 2016. Table 4 presents key informant participant characteristics.

**TABLE 4:** Characteristics of the key informant participants.

	Frequency
**Gender**	
Female	13
Male	2
**Highest level of education**	
Post‐graduate degree	7
Baccalaureate degree	4
Doctor of Medicine	2
College diploma	1
Two post‐graduate degrees	1
**Profession/occupation**	
Registered nurse	5
Physician	4
Nurse practitioner	2
Social worker	2
Spiritual health practitioner	1
Licensed practical nurse	1
**Role related to MAID and end‐of‐life care**	
Only a MAID service team member	8
Member of palliative care and MAID service	4
Facilitator of psychosocial support services	2
Only a member of palliative care and hospice service	1
**When became connected to MAID**	
Before implementation (prior to June 2016)	7
During first year of legalization (July 2016 to June 2017)	4
At the time of implementation (June 2016)	3
Since July 2018	1

*Note:* This table outlines the characteristics of the key informant sample.

### 5.2 Understanding the Context

Family members' experiences of MAiD were shaped by more than the decision itself. Our analysis indicated that family members' comprehension of the death, emotional preparation, and support for their relative were influenced by three contextual elements: (1) *Knowing the Perspective*, (2) *Being Aware of the Suffering,* and (3) *Managing Personal Beliefs*. Collectively, these elements informed family members' understanding of the MAiD decision, their responses to moral tensions, and their experiences of grief and bereavement.

Family members' understanding of their relative's perspective influenced how they interpreted suffering, while personal beliefs shaped how they reconciled the decision with their own values. Our interpretation suggests that the relationship among these contextual elements contributed to the complexity and variability of family members' experiences with MAiD.

#### 5.2.1 Knowing the Perspective

Our analysis positioned understanding the perspective of the individual choosing MAiD as a key contextual element influencing family members' emotional preparedness and confidence in the decision. Prior awareness of a relative's views on autonomy and end‐of‐life choices provided a framework for interpreting the decision and preparing for MAiD.

When participants had longstanding knowledge of their relative's views, they perceived MAiD as consistent with previously expressed values rather than as an unexpected decision. One daughter noted that MAiD was “always on our radar… Long before it became legal, she had talked about it” (FM‐17). This consistency appeared to reduce shock and uncertainty, enabling family members to assume supportive roles more readily.

For other participants, awareness developed gradually through diagnosis, illness progression, advance care planning, and conversations about end‐of‐life wishes. These experiences incorporated MAiD into broader end‐of‐life preparation and, for some, eased emotional tension. One participant described how their mother actively planned for the end‐of‐life, including arranging “treasure hunts” to locate organizing documents and medical directives (FM‐22). Such planning situated MAiD within an ongoing expression of agency rather than as a sudden decision.

In contrast, participants who learned of their relative's intention to pursue MAiD only shortly before the decision described greater emotional challenges. Limited time provided fewer opportunities to process the decision, reconcile personal values, or address unresolved relational concerns. One participant described feeling “hit by a ten‐tonne truck” (FM‐05), conveying both the shock and the limited time to adjust. Our interpretation suggests that limited prior knowledge appeared to constrain family members' opportunities to understand the decision and prepare for the MAiD event. Clinically, nurses can support earlier conversations about end‐of‐life values and goals of care, while respecting the wishes and autonomy of the individual accessing MAiD. When family involvement is appropriate and desired, anticipatory guidance may help family members prepare for the emotional and practical complexities associated with a relative's decision to pursue MAiD.

#### 5.2.2 Being Aware of the Suffering

Our interpretation suggests that awareness of suffering was a contextual element through which family members understood and came to accept, or reconcile with, their relative's decision to pursue MAiD. Participants described suffering not only in terms of physical or functional decline but also as a factor shaping their understanding of the decision.

For many participants, witnessing ongoing deterioration transformed MAiD from an abstract possibility into a response they could understand in relation to their relative's suffering. Descriptions such as being “a shell of a human” (FM‐16) illustrated how visible decline shaped perceptions of MAiD as an act of care rather than solely as a death. In this context, witnessing suffering seemed to help family members reconcile their responses with the individual's decision to receive MAiD.

For some family members, this understanding developed gradually, particularly in the context of progressive, life‐limiting conditions. Observable decline made the rationale for MAiD easier to understand. Participants described becoming “one hundred percent behind” (FM‐26) the person choosing MAiD as suffering intensified. This suggests that proximity to decline contributed to greater acceptance by making suffering more tangible and difficult to deny.

Conversely, when family members were geographically distant or had limited exposure to the person choosing MAiD and their suffering, understanding the decision was often more challenging. Limited contact appeared to contribute to disbelief or resistance. One participant described how her son “didn't believe [his dad] was dying” and began staying overnight to experience how his dad was managing (FM‐23). Without direct exposure to the decline, suffering, or tangible evidence, deciding for MAiD was harder for some family members to reconcile.

Our interpretation suggests that proximity to and visibility of suffering influenced how family members understood and responded to the decision to pursue MAiD. Clinically, nurses can support family members in developing a shared understanding of the illness trajectory and quality of life, particularly when family members have limited opportunities to witness or discuss changes in the individual's condition.

#### 5.2.3 Managing Personal Beliefs

Our analysis positioned personal beliefs as a contextual element through which family members negotiated tensions between their own values and their commitment to respecting their relative's autonomy. Rather than solely determining acceptance or opposition, beliefs influenced how family members interpreted and responded to the decision to pursue MAiD.

For some participants, alignment between personal beliefs and the principles underlying MAiD facilitated acceptance with reduced internal conflict. Participants who approached MAiD from pragmatic or secular perspectives described it as a logical extension of humane care (FM‐01).

Other participants reported internal struggle but chose to set aside their uneasiness to avoid influencing their relative's decision. Avoiding “seeds of doubt” (FM‐06) reflected a commitment to respect autonomy, even when this was emotionally and psychologically difficult. These accounts indicate that outward support for MAiD can coexist with significant internal conflict.

When family members' beliefs diverged from those of the individual accessing MAiD, emotional strain sometimes intensified and persisted into bereavement. For example, one participant described urging her sister to say goodbye to their father despite ongoing disagreement with the decision (FM‐25), illustrating how belief differences could complicate relationships and support. Even among family members who actively supported the decision, unresolved moral tensions remained. One husband described his experience as “murder” (FM‐02) despite his active involvement in caring for his wife and participating in the MAiD process, highlighting the depth of conflict that could persist beyond the death.

Our interpretation suggests that managing personal beliefs shaped both family members' involvement during the MAiD process and their experiences of bereavement. Taken together, *Knowing the Perspective*, *Being Aware of the Suffering* and *Managing Personal Beliefs* provide an interpretive account of how family members make sense of and respond to their relative's decision to pursue MAiD. Clinically, attending to family members' understanding of the individual's end‐of‐life values, exposure to suffering, and sources of moral tension may support timely, relationally responsive care while respecting the autonomy of the person accessing MAiD.

### 5.3 Overarching Themes to Understanding the Experience

In addition to contextual elements influencing family members' experiences, our analysis developed three overarching interpretive themes that describe how family members experienced and responded to their relative's or close person's journey toward MAiD: (a) *They want MAiD; now what?* (b) *Prepared but maybe not ready,* and (c) *Evolving understanding of this type of death*. Through iterative interpretation of participants' accounts, these themes reflect how family members prepare for, participate in, and make sense of MAiD within personal, relational and healthcare contexts. Several subthemes further elaborate these interpretations.

Together, these themes illustrate how family members navigated unfamiliar healthcare processes, assumed emotionally and practically complex roles, and developed an understanding of death distinct from what they anticipated or usually experience. Collectively, these interpretations extend an autonomy‐focused perspective by highlighting its relational, ethical and emotional implications for those who remain after the death.

#### 5.3.1 They Want MAiD, Now What?

When a relative expressed a desire to pursue MAiD, family members described a shift in their caregiving role. They shifted from providing care and witnessing the individual's decline to navigating unfamiliar healthcare processes, including interpreting information, advocating for access and coordinating care. Our analysis indicated that healthcare system processes, available services, and responsiveness influenced family members' distress, confidence and sense of support.

##### 5.3.1.1 Navigating Access

Family members reported varying degrees of involvement and responsibility when navigating MAiD access for their relative. Program and service clarity, along with healthcare professional responsiveness, shaped these experiences. When MAiD processes were clear and healthcare professionals demonstrated knowledge and compassion, family members perceived the system as integrated and supportive. This reduced the need for advocacy and system navigation. As one participant recalled, “That hospital was absolutely stellar… The staff were the most amazingly compassionate, empathetic people I have ever met” (FM‐18).

Even when healthcare professionals had limited experience with MAiD, family members reported reassurance when clinicians remained engaged and communicative. One participant described seeking guidance from a social worker who, despite initial uncertainty, actively followed up and “walked me through it [MAiD request paperwork]…I needed somebody to say the words” (FM‐14). This interaction offered both procedural guidance and relational support during a stressful period.

In contrast, unclear institutional processes and limited information heightened distress for some family members. One participant described being denied information by hospice staff and perceived that their questions were viewed as disruptive, “I expected better… I think my sister and I were causing angst because of our questions and assertiveness” (FM‐13). Such experiences contributed to frustration and disappointment, particularly when they differed from previous supportive interactions within the healthcare system.

Our interpretation positions access to MAiD as a relational process rather than solely an administrative one. In clinical contexts, knowledgeable and responsive healthcare professionals, combined with transparent organizational guidance, may reduce the burden of navigating MAiD for family members. Conversely, ambiguous or restrictive processes can shift responsibility for navigating access, including obtaining and submitting the MAiD request, onto family members at times when clarity and support are especially critical.

##### 5.3.1.2 Appreciating Palliative Care

Many family members indicated that palliative care was in place before their relative made a formal MAiD request. However, several participants described a perceived decline in palliative care engagement once MAiD became part of the care trajectory, “Basically, the palliative team disappeared once [he] applied for MAiD” (FM‐07). This shift was especially apparent as their relative's care needs increased.

Some family member participants expressed disappointment that MAiD and palliative care appeared to function as distinct rather than integrated services. As one participant reflected, “At the beginning, I thought MAiD would be linked with palliative care… It saddens me. I assumed they would go hand in hand” (FM‐27). Participants described this perceived discontinuity as placing additional responsibility on family members and increasing their burden. Family members also reported limited access to emotional and bereavement support. Few participants described sustained palliative care involvement as their relative approached the MAiD event.

Key informants also described the value of integrating palliative care and MAiD. One MAiD provider emphasized the contribution of palliative care nurses to family support beyond the MAiD procedure, “They give all the grief support, stay with the family, wait for the funeral home. If it's another nurse, you don't get that” (KI‐11). This account illustrates how continuity across services can support family members before, during and after the MAiD event.

Our interpretation positions continuity between MAiD and palliative care as an important influence on family members' experiences. When services were perceived as integrated, family members reported greater access to support. Conversely, reduced engagement meant family members took on more responsibility for navigating care and the healthcare system. These findings highlight the importance of coordinated models of care that support both individuals accessing MAiD and their family members throughout the care trajectory.

#### 5.3.2 Prepared but Maybe Not Ready

Many family members described feeling practically prepared for MAiD while remaining emotionally uncertain. Their involvement could be meaningful and affirming while also creating emotional and moral strain. As involvement intensified, family members reflected on their responsibilities, their potential influence on the decision, and whether they had done enough. These questions sometimes persisted into bereavement.

##### 5.3.2.1 Reconciling Involvement

Family members described involvement in a relative's pursuit of MAiD as dynamic, often intensifying as the process unfolded. For some, involvement grew out of established caregiving roles; for others, MAiD required new personal, emotional and logistical commitments. As their involvement increased, some family members interpreted their actions through a moral lens, experiencing guilt and self‐scrutiny despite actively supporting the decision. One participant reflected, “felt guilt. I felt that if I could have done something more… or if I had been able to have her live with me [maybe she would have chosen to live longer]” (FM‐17).

As the MAiD date approached, family members described both increased emotional closeness and uncertainty about whether their support had influenced the decision. One son expressed this internal conflict, “I was scared… maybe she would be influenced, like she was influenced by me to get approved. I remember feeling a lot of turmoil inside” (FM‐22). Our interpretation suggests that family members felt more reassured when they understood the decision to be autonomous and firmly held by the person choosing MAiD.

Involvement took on psychological significance as death approached and continued into their bereavement. Family members reflected on whether they had fulfilled their responsibilities and “done enough” (FM‐20). One daughter stated, “I keep having to remind myself that I did my best… it was good to hear that [my mother] knew how much I was doing for her” (FM‐20). For some, a sense of having done enough helped reconcile their involvement and shaped how they understood their experience in bereavement.

For others, involvement was distressing, particularly when it included facilitating a MAiD request or access to MAiD services. One husband described this shift in perspective, “The actual act of killing her, which is what it is, was a total shock… I am not an accessory after the fact; I am a participant” (FM‐02). This account illustrates how involvement could blur the boundaries between supporting, facilitating and feeling morally responsible for the death.

Our interpretation highlights that family involvement in MAiD extends beyond caregiving and advocacy to include ethical, emotional and identity‐related dimensions. Family members may experience themselves as both supporters of the decision and morally implicated in the process. This contributes to the emotional complexity of their experience, which can persist into bereavement.

##### 5.3.2.2 Living in Suspension

Family members described periods of uncertainty and limited control while awaiting eligibility determinations and navigating logistical aspects under Bill C‐14. Our interpretation suggests that this waiting disrupted emotional preparation and contributed to feelings of helplessness as family members anticipated their relative's death.

The waiting periods were emotionally taxing and destabilizing for family members. As one daughter explained, “Mom's assessments took almost a week to set up, and they were 3 days apart… We didn't know [that she qualified], and then it was close to [the date chosen for MAiD]” (FM‐14). Uncertainty about eligibility constrained family members' ability to prepare emotionally, leaving them feeling unable to move forward while aware that their time with their relative might be limited.

Key informants offered additional context on the mismatch between procedural timelines and family members' perceptions of time. One healthcare provider noted, “Time runs on a different scale… for the family it's like five or 10 times longer than the actual clock time” (KI‐05). This account supports our interpretation that waiting is experienced as a prolonged emotional strain, rather than just a procedural process or a delay, during an already challenging period.

For some family members, the challenges associated with waiting were intensified by healthcare teams' lack of familiarity with MAiD processes during early implementation. One daughter recalled being asked to “bear with us while we try to figure this out”, describing the experience as particularly stressful during an already upsetting period (FM‐04). In these circumstances, family members experienced uncertainty and assumed responsibility without corresponding agency or authority.

Our interpretation highlights uncertainty as a significant factor influencing family members' emotional readiness and sense of agency. Clinically, nurses and other healthcare professionals may help reduce uncertainty by providing regular updates, explaining procedural steps and anticipated timelines, and designating a consistent point of contact for family members throughout the MAiD process.

##### 5.3.2.3 Realizing the End

Although family members generally supported their relative's decision to pursue MAiD, scheduling the date of death elicited profound and complex emotional responses. These reactions did not necessarily reflect opposition to MAiD but rather the psychological weight of knowing when their relative would die. As one son observed, “Death is always a random thing. When you say, the 18th of October, then it's not random anymore. It's very tough” (FM‐30). Others described the experience as putting “a pin in your heart” (FM‐29) or feeling “unnatural… like my brain could not process it” (FM‐16).

Many participants delayed attending to their own emotions while focusing on the needs of the person accessing MAiD. One wife explained, “I didn't give it [MAiD] much attention… I was obviously trying to justify any way to keep him around with me longer” (FM‐16). Confirmation of the date marked a turning point for some, making the impending death more immediate, “This time next week, Dad's not going to be here… Yeah, I do know when he's going to die. So strange” (FM‐19).

For some, the scheduling of the MAiD event also felt impersonal and disconnected from its emotional significance. One participant compared the process to “booking a pizza… like we are talking to an airline booking agent” (FM‐02). The contrast between the administrative nature of scheduling and the profound significance of the impending death left some family members feeling their emotional needs were not fully recognized.

Our interpretation positions scheduling the date of death as a relational and emotional transition, not solely an administrative activity. Recognizing this transition may help nurses and other healthcare professionals anticipate family members' emotional responses. Clinically, nurses can create opportunities for questions, reflection and support before and after the MAiD event.

#### 5.3.3 Evolving Understanding About This Type of Death

Family members characterized MAiD as enabling a distinct and intentional death experience, one that created opportunities for presence, connection and deliberate goodbyes. Although MAiD enabled meaningful farewells, it also raised questions about how the death was understood, disclosed and remembered. Our interpretation suggests that these experiences shaped family members' emotional preparation, relational connections and bereavement.

##### 5.3.3.1 Enabling Celebration, Reminiscing and Saying Goodbye

Despite the emotional weight of an assisted death, many family members characterized the period leading up to the MAiD event as meaningful and relationally rich. Knowing the date and having time with the individual accessing MAiD enabled intentional acts of remembering, celebration and farewell. Participants recounted looking through photo albums, preparing favourite meals and gathering with others to honour the individual. One daughter reflected, “It's that idea of people having their funeral before they die so they can hear all the good things people say about them… [We] had that experience, and it was lovely” (FM‐28).

Planning personal rituals and symbolic events was described as particularly meaningful, allowing family members to mark milestones that might otherwise have been missed. One participant described hosting a small celebration so their mother could participate in the engagement, noting, “My mom wrote a toast to us” (FM‐22). Such experiences fostered opportunities for connection and helped family members acknowledge the impending death.

Family members described experiences on the day of the MAiD event as intimate and embodied, reinforcing a sense of closeness and presence. A son shared, “I laid on her chest until I couldn't hear her heartbeat anymore” (FM‐20), while another participant described the atmosphere as “basically a love orgy of the five of us all together” (FM‐30). Participants expressed gratitude for being present, saying goodbye while their relative retained capacity and experiencing the death together. We interpret these experiences as important to how family members prepared for and entered bereavement. From a clinical perspective, nurses can support family members by recognizing opportunities for legacy‐making, meaningful rituals, and connection before the MAiD event.

##### 5.3.3.2 Keeping the Type of Death Private

Alongside meaningful experiences, some family members intentionally maintained privacy about the circumstances of the death. Although MAiD enabled open conversations about values, death, and dying for some family members, disclosure could be selective. Our interpretation suggests that privacy was an ongoing relational process influenced by concerns about stigma, judgment, and potentially disruptive interactions. For example, one participant withheld information from distant relatives until after the death, explaining, “They'd have called and made a big deal. We just phoned them after it was finished” (FM‐32). For these family members, maintaining privacy helped maintain a sense of control and calm during a highly personal period.

Concerns about protest, public scrutiny, and moral judgement also influenced disclosure. One spouse recounted, “I told them they couldn't tell anyone because I didn't want anyone protesting on my front lawn” (FM‐13). For others, caution extended to immediate family members, “My son still doesn't know… I have to feel out how he feels about MAID” (FM‐19). Participants described these decisions as protective measures intended to safeguard relationships and preserve their relative's memory.

Religious and community contexts also influenced these decisions. One family member shared, “I didn't talk about it much… I was worried it would be frowned on” (FM‐08). A key informant noted that some family members appeared to attribute the death to the underlying medical condition rather than MAiD to reduce the chance of judgment (KI‐01).

Our interpretation highlights that concerns about stigma and disclosure can extend into bereavement, shaping how family members communicate about the death. Clinically, nurses can create non‐judgemental opportunities to discuss disclosure concerns and connect family members with MAiD‐informed psychosocial and bereavement supports.

## 6 Discussion

This study explored how bereaved family members navigate and interpret the experience when a relative or close person receives MAiD in Canada. Our analysis highlights the relational, emotional, and practical dimensions of family involvement and offers practice‐relevant insights for nursing and clinical practice.

Understanding a relative's values, whether longstanding or evolving with illness, was central to how family member participants approached MAiD. This understanding provided a relational context for preparedness and acceptance, even when emotional ambivalence remained. Previous research demonstrates that proximity to suffering influences family members' understanding of MAiD (Beuthin et al. 2021). Our analysis suggests that witnessing suffering may make a relative's decision to pursue MAiD more comprehensible and affirm family members' supportive roles.

Family members' moral positions varied, with some experiencing ongoing internal conflict. Many participants emphasized respect for the autonomy of the person accessing MAiD and understood their involvement as a commitment to honouring that person's wishes. Previous studies have described caregiving and emotional support in MAiD contexts (Brown et al. 2020; Holmes et al. 2018; Thangarasa et al. 2021). Our analysis extends this knowledge by positioning family involvement as relational solidarity. This involvement may include emotional, practical and moral responsibilities. For some, the internal conflict and tension persisted into bereavement, reflecting the enduring complexity of making sense of a MAiD death (Frolic et al. 2020; Serota et al. 2024).

Family members also assumed active roles in navigating MAiD processes and services, including coordinating logistics and advocating for access. These responsibilities intensified when healthcare professionals were perceived as hesitant, unclear, or disengaged. Fragmented information and communication contributed to emotional and logistical burdens, consistent with research identifying system‐related stressors in MAiD care (Brown et al. 2020; Close et al. 2023; Crumley et al. 2023; Rumsey et al. 2026). These gaps sometimes left family members navigating the healthcare system and MAiD services with limited guidance or support.

Most family member participants reported that their relatives received palliative care before initiating MAiD. However, some questioned whether the quality and consistency of palliative care influenced the timing of the MAiD decision, reflecting broader concerns about access to high‐quality palliative care in Canada (Canadian Association of MAiD Assessors and Providers, n.d.). Additionally, several participants also described reduced involvement from palliative care after a MAiD request. This separation between palliative and MAiD care may create a gap in support at a time when both the individual and their family members have increasing needs. Integrated approaches and greater continuity between palliative care and MAiD services may help address this gap.

Despite being practically prepared, many family members experienced emotional upheaval, including guilt and self‐doubt (Crumley et al. 2023). Participants questioned whether they had influenced their relative's decision or failed to advocate for additional care options. Even those who supported MAiD described emotional complexity that continued into bereavement (Frolic et al. 2020; Hales et al. 2019). Family members may therefore benefit from psychosocial support before and after a MAiD death (Beuthin et al. 2021; Brown et al. 2020; Smolej et al. 2022). Active involvement in the MAiD process should not be assumed to mean that family members are emotionally prepared for the death or subsequent bereavement.

Knowing the precise date and time of death also carried significant emotional weight (Beuthin et al. 2021; Frolic et al. 2020; Serota et al. 2024). Although this certainty created opportunities for connection and preparation, it could also intensify anticipatory grief (Crumley et al. 2023; Oczkowski et al. 2021). Several participants focused on supporting their relative and ensuring the process proceeded smoothly, often postponing attention to their own emotional needs. For nurses and other healthcare professionals, this underscores the importance of creating space for family members to attend to their own emotions before the MAiD event, rather than focusing solely on the process's practicalities.

Scheduling the event also emerged as an underexplored relational dimension of MAiD. Although the decision rested with the person accessing MAiD, family members sometimes experienced scheduling as shaped by logistical constraints and provider availability. Several described interactions with healthcare professionals as transactional. Recognizing the emotional significance of scheduling the MAiD event may create opportunities for more relationally responsive care.

Consistent with previous research, some family members maintained privacy around MAiD because of perceived stigma, cultural or religious concerns, or the wishes of the person accessing MAiD (Close et al. 2023; Fortin et al. 2026; Frolic et al. 2020). For some, this silence continued into bereavement, which contributed to emotional strain (Hales et al. 2019). At the same time, participants described meaningful opportunities for connection, reminiscence and closure before the death (Beuthin et al. 2021). Socio‐cultural constraints sometimes limited these opportunities and influenced how the death was understood and remembered.

Interactions with healthcare professionals also influenced family members' experiences. When clinicians demonstrated knowledge, approachability and relational responsiveness, family members felt acknowledged and supported. In contrast, perceived judgment or disengagement heightened stress and isolation, consistent with previous findings (Brown et al. 2020; Close et al. 2023; Rumsey et al. 2026). Our analysis suggests that supporting family members requires more than knowledge of MAiD processes. Nurses and other healthcare professionals also need to be willing to openly engage in discussions, seek information when needed, and attend to family members' emotional and relational needs.

Although MAiD legislation centers on individual autonomy, our interpretation frames MAiD as both an individual and relational experience, particularly for those who remain after the death. Family members contributed to the MAiD journey by interpreting values, navigating systems, and carrying emotional and moral consequences, often with limited formal support. Attending to these experiences makes visible the relational consequences of MAiD for those who accompany the person through the process and live with the death afterward.

### 6.1 Implications for Nursing Practice: A Family‐In‐Focus Approach

Based on these findings, we proposed a family‐in‐focus approach to MAiD. This approach does not replace patient‐centred care but extends it by explicitly recognizing the relational context surrounding the person accessing MAiD. Family members may appear organized and supportive while experiencing emotional upheaval, moral uncertainty and deferred grief.

A family‐in‐focus approach calls for nurses to engage family members proactively, normalize emotional complexity, and provide anticipatory guidance when they are involved in a person's MAiD journey. Practical strategies may include family check‐ins, clear and consistent communication about processes and timelines, and access to MAiD‐specific psychosocial and bereavement supports. Support should begin when MAiD becomes part of the care trajectory and continue through the MAiD event and into bereavement. This approach keeps the person accessing MAiD central while ensuring that involved family members remain visible within the frame of care.

### 6.2 Strengths and Limitations

A strength of this study is its focus on bereaved family members whose relative or other close person received MAiD during the first 5 years of its implementation in Canada (2016–2020). Using Interpretive Description and multiple data sources yielded practice‐relevant insights into the emotional, logistical and relational aspects of family members' experiences. Key informants, documents, artifacts, field notes, and analytic memos provided clinical, organizational, and system‐level context. Our findings highlight that emotional and moral tensions may persist even among family members who support MAiD. This finding underscores the importance of nursing care that addresses family members' needs alongside those of the individual accessing MAiD. These insights informed a family‐in‐focus approach that increases the visibility of family members within the MAiD care trajectory.

Study limitations include limited sample diversity. Most participants were female, lived in urban areas, and were generally supportive of MAiD. Self‐selection bias may also have influenced participation. The key informant group did not include policymakers or senior healthcare leaders, limiting system‐level perspectives. Data collection occurred during COVID‐19 public health measures and may have excluded individuals with limited internet access or those less comfortable with technology. Data were collected under Bill C‐14 and therefore do not reflect experiences under Bill C‐7, which expanded eligibility to individuals whose natural death was not reasonably foreseeable (Nicol and Tiedemann 2021). Excluding family members of individuals who used self‐administered MAiD also limits the applicability of the findings to this less frequently used form of MAiD. Finally, variation in bereavement length may have influenced participants' recall and framing of their experiences.

### 6.3 Recommendations for Further Research

Future research should examine the experiences of family members in rural and remote areas, including how resource availability, privacy concerns and access to healthcare shape experiences of MAiD. Further research is also needed on the influence of religion and spirituality, the long‐term implications of selective secrecy and disclosure, and experiences of family members who did not support a relative or close person's decision. As MAiD legislation and practice continue to evolve, research on family members' experiences in Track 2 cases and the intersection between palliative care and MAiD would be valuable. Further investigation into nurses' roles in providing anticipatory guidance, supporting family members involved in MAiD, and bereavement care is also needed. Including family members connected to self‐administered MAiD would address another gap in the literature. Finally, future studies could explore methods to operationalize and evaluate a family‐in‐focus approach across diverse MAiD settings and care contexts.

## 7 Conclusion

This Interpretive Description study explored how bereaved family members experience the death of a relative or close person through MAiD. Family members' experiences were shaped by their understanding of the individual's values, proximity to suffering, and negotiation of personal moral beliefs. These conceptual elements influenced how family members interpreted the decision, supported the individual accessing MAiD, and prepared for the impending death. The findings expand predominantly procedural and autonomy‐focused perspectives on MAiD by highlighting its relational, emotional and practical dimensions for surviving family members. Family members assumed practical and emotional responsibilities, including navigating MAiD processes and advocating for the individual accessing MAiD. Many described meaningful farewells and close relational connections while also experiencing uncertainty, moral strain and self‐scrutiny. Supporting an individual's decision to pursue MAiD did not necessarily correspond to emotional preparedness for their death or subsequent bereavement.

These findings support a family‐in‐focus approach to MAiD that complements, rather than replaces, person‐centred care. Proactive communication, recognition of emotional complexity, and access to integrated psychosocial and bereavement support are key elements of this approach. Nurses and other healthcare professionals can enhance the visibility of family members throughout the MAiD care trajectory while upholding the autonomy of the individual accessing MAiD. Addressing both dimensions can facilitate care that respects the individual's right to choose and acknowledges the relational, emotional and practical experiences of those who accompany them.

## Author Contributions

**Tracy L. Powell:** conceptualization, data curation, formal analysis, funding acquisition, investigation, methodology, project administration, writing – original draft, writing – review and editing. **Kelli Stajduhar:** conceptualization, methodology, formal analysis, supervision, validation, writing – review and editing, investigation. **Michael Prince:** conceptualization, writing – review and editing, validation, methodology, formal analysis, supervision. **Sally Thorne:** conceptualization, methodology, formal analysis, supervision, validation, writing – review and editing.

## Funding

This research received funding from Mount Royal University (no. 101677 and 102316) through an Internal Research Grant from the Office of Research, Scholarship, and Community Engagement, and an Innovation Grant from the Faculty of Health, Community, and Education.

## Ethics Statement

This study was reviewed and approved by the Harmonized University of Victoria Health Research Ethics Board, the Health Research Ethics Board of Alberta—Community Health Committee, and the Human Research Ethics Board at Mount Royal University (Study Number: BC19‐0391).

## Conflicts of Interest

The authors declare no conflicts of interest.

## Data Availability

The data that support the findings of this study are available from the corresponding author upon reasonable request.

## References

[nop270843-bib-0001] Arnup, K. 2018. “Family Perspectives: Death and Dying in Canada.” http://vanierinstitute.ca/deathdying2018/.

[nop270843-bib-0002] Austin, W. , V. Bergum , and J. Dossetor . 2003. “Relational Ethics: An Action Ethic as a Foundation for Health Care.” In Approaches to Ethics: Nursing Beyond Boundaries, edited by V. Tschudin , 45–52. Butterworth‐Heinemann.

[nop270843-bib-0003] Bentley, B. , and M. O'Connor . 2015. “Conducting Research Interviews With Bereaved Family Carers: When Do We Ask?” Journal of Palliative Medicine 18, no. 3: 241–245. 10.1089/jpm.2014.0320. PMC4348368 25517136

[nop270843-bib-0004] Bergum, V. , and J. Dossetor . 2005. Relational Ethics: The Full Meaning of Respect. University Publishing Group.

[nop270843-bib-0005] Beuthin, R. , A. Bruce , M. Thompson , A. E. B. Andersen , and S. Lundy . 2021. “Experiences of Grief‐Bereavement After a Medically Assisted Death in Canada: Bringing Death to Life.” Death Studies 46, no. 8: 1982–1991. 10.1080/07481187.2021.1876790. 33538647

[nop270843-bib-0006] Brown, J. , D. Goodridge , A. Harrison , J. Kemp , L. Thorpe , and R. Weiler . 2020. “Care Considerations in a Patient‐ and Family‐Centered Medical Assistance in Dying Program.” Journal of Palliative Care 37, no. 3: 341–351. 10.1177/0825859720951661. 32854581

[nop270843-bib-0007] Canadian Association of MAID Assessors and Providers . n.d. “Palliative Care & MAiD.” https://camapcanada.ca/clinicians/clinician‐resources/pallative‐care‐maid/.

[nop270843-bib-0008] Close, E. , R. Jeanneret , J. Downie , L. Willmott , and B. P. White . 2023. “A Qualitative Study of Experiences of Institutional Objection to Medical Assistance in Dying in Canada: Ongoing Challenges and Catalysts for Change.” BMC Medical Ethics 24, no. 71: 1–24. 10.1186/s12910-00950-9. PMC10512474 37735387

[nop270843-bib-0009] Crumley, E. T. , J. LeBlanc , B. Henderson , C. S. Jackson‐Tarlton , and E. Leck . 2023. “Canadian Family Members' Experiences With Guilt, Judgment and Secrecy During Medical Assistance in Dying: A Qualitative Descriptive Study.” CMAJ Open 11, no. 4: E782–E789. 10.9778/cmajo.20220140. PMC10449018 37607750

[nop270843-bib-0010] Fortin, S. , S. Lessard , and M. Samson . 2026. “Diversity and Access to Palliative Care and Medical Assistance in Dying in an Urban Setting.” Omega–Journal of Death and Dying 93, no. 1: 297–316. 10.1177/0030228241237834. PMC13002951 38448056

[nop270843-bib-0011] Frolic, A. N. , M. Swinton , L. Murray , and A. Oliphant . 2020. “Double‐Edged MAiD Death Family Legacy: A Qualitative Descriptive Study.” BMJ Supportive & Palliative Care 14: e845–e850. 10.1136/bmjspcare-2020-002648. 33355177

[nop270843-bib-0012] Gamondi, C. , M. Pott , N. Preston , and S. Payne . 2018. “Family Caregivers' Reflections on Experiences of Assisted Suicide in Switzerland: A Qualitative Interview Study.” Journal of Pain and Symptom Management 55, no. 4: 1085–1094. 10.1016/j.jpainsymman.2017.12.482. 29288877

[nop270843-bib-0013] Hales, B. M. , B. Ford , S. Bean , E. Isenberg‐Grzeda , and D. Selby . 2019. “Improving the Medical Assistance in Dying (MAID) Process: A Qualitative Study of Family Caregiver Perspectives.” Palliative & Supportive Care 17, no. 5: 590–595. 10.1017/S147895151900004X. 30887936

[nop270843-bib-0014] Handique, S. , S. Jang , M. Bennett , and S. D. Ryan . 2025. “Invisible Pillars With Hidden Burdens: A Scoping Review on the Challenges of Informal Caregivers for Older Adults in Home‐Based Palliative Care.” Journal of Palliative Care 41, no. 2: 186–196. 10.1177/08258597251348262. PMC13013662 40509572

[nop270843-bib-0015] Hashemi, N. , E. Amos , and B. Lokuge . 2021. “The Quality of Bereavement for Caregivers of Patients Who Died by Medical Assistance in Dying at Home and the Factors Impacting Their Experience: A Qualitative Study.” Journal of Palliative Medicine 24, no. 9: 1351–1357. 10.1089/jpm.2020.0654. 33513049

[nop270843-bib-0016] Health Canada . 2024. “Fifth Annual Report on Medical Assistance in Dying in Canada, 2023.” https://www.canada.ca/en/health‐canada/services/publications/health‐system‐services/annual‐report‐medical‐assistance‐dying‐2023.pdf.

[nop270843-bib-0017] Holmes, S. , E. R. Wiebe , J. Shaw , A. Nuhn , A. Just , and M. Kelly . 2018. “Exploring the Experience of Supporting a Loved One Through a Medically Assisted Death in Canada.” Canadian Family Physician 64, no. 9: e387–e393. PMC6135137 30209112

[nop270843-bib-0018] Krawczyk, M. , R. Sawatzky , K. Schick‐Makaroff , et al. 2019. “Micro‐Meso‐Macro Practice Tensions in Using Patient‐Reported Outcome and Experience Measures in Hospital Palliative Care.” Qualitative Health Research 29, no. 4: 510–521. 10.1177/1049732318761366. 29542400

[nop270843-bib-0019] Morse, J. M. 1994. “‘Emerging From the Data’: The Cognitive Processes of Analysis in Qualitative Inquiry.” In Critical Issues in Qualitative Research Methods, edited by J. M. Morse , 23–45. SAGE.

[nop270843-bib-0020] National Alliance for Caregiving . 2009. “The Economic Downturn and Its Impact on Family Caregiving.” https://www.caregiving.org/wp‐content/uploads/2020/05/EVC_Caregivers_Economy_Report‐FINAL_4‐28‐09.pdf.

[nop270843-bib-0021] Nicol, J. , and M. Tiedemann . 2021. Legislative Summary of Bill C‐7: An Act to Amend the Criminal Code (Medical Assistance in Dying) (Publication No. 43‐2‐C7‐E). Library of Parliament. https://lop.parl.ca/sites/PublicWebsite/default/en_CA/ResearchPublications/LegislativeSummaries/432C7E.

[nop270843-bib-0022] Oczkowski, S. J. W. , D. E. Crawshaw , P. Austin , et al. 2021. “How Can We Improve the Experiences of Patients and Families Who Request Medical Assistance in Dying? A Multi‐Centre Qualitative Study.” BMC Palliative Care 20: 185. 10.1186/s12904-021-00882-4. PMC8653618 34876104

[nop270843-bib-0023] Patton, M. Q. 2015. Qualitative Research and Evaluation Methods. 4th ed. SAGE.

[nop270843-bib-0024] Rumsey, C. , D. W. McKennitt , T. Stafinski , and D. Menon . 2026. “Family Perspectives on High‐Quality MAiD Service and Delivery: A Cross‐Canada Qualitative Description Study.” BMC Palliative Care 25, no. 62: 1–16. 10.1186/s12904-026-02035-x. PMC12983891 41781964

[nop270843-bib-0025] Sandelowski, M. 1995. “Sample Size in Qualitative Research.” Research in Nursing & Health 18, no. 2: 179–183. 10.1002/nur.4770180211. 7899572

[nop270843-bib-0026] Serota, K. , M. Atkinson , and D. Z. Buchman . 2024. “Mapping MAiD Discordance: A Qualitative Analysis of the Factors Complicating MAiD Bereavement in Canada.” Qualitative Health Research 34, no. 3: 195–204. 10.1177/10497323231208540. PMC10768327 37972933

[nop270843-bib-0027] Smolej, E. , M. Malozewski , S. McKendry , et al. 2022. “A Qualitative Study Exploring Family Caregivers' Support Needs in the Context of Medical Assistance in Dying.” Palliative & Supportive Care 21, no. 2: 254–260. 10.1017/s1478951522000116. 35642744

[nop270843-bib-0028] Statistics Canada . 2018. “General Social Survey—Caregiving and Care Receiving (GSS).” http://www23.statcan.gc.ca/imdb/p2SV.pl?Function=getSurvey&SDDS=4502.

[nop270843-bib-0029] Thangarasa, T. , S. Hales , E. Tong , et al. 2021. “A Race to the End: Family Caregivers' Experience of Medical Assistance in Dying (MAiD)—A Qualitative Study.” Journal of General Internal Medicine 37: 809–815. 10.1007/s11606-021-07012-z. PMC8904693 34287775

[nop270843-bib-0030] Thorne, S. 2016. Interpretive Description: Qualitative Research for Applied Practice. 2nd ed. Routledge.

[nop270843-bib-0031] Wright, D. , S. Brajtman , and M. E. Macdonald . 2018. “Relational Ethics of Delirium Care: Findings From a Hospice Ethnography.” Nursing Inquiry 25, no. 3: 1–10. 10.1111/nin.12234. 29573054

